# Emerging roles and therapeutic value of exosomes in cancer metastasis

**DOI:** 10.1186/s12943-019-0964-8

**Published:** 2019-03-30

**Authors:** Miaowei Wu, Guosheng Wang, Weilei Hu, Yihan Yao, Xiao-Fang Yu

**Affiliations:** 1 Cancer Institute (Key Laboratory of Cancer Prevention and Intervention, Ministry of Education), Second Affiliated Hospital, School of Medicine, Zhejiang University, Hangzhou, 310009 People’s Republic of China; 2 Inst Translat Med, School of Medicine, Zhejiang University, Hangzhou 310029 Zhejiang, People’s Republic of China; 3 Department Surg Oncol, Second Affiliated Hospital, School of Medicine, Zhejiang University, Hangzhou, 310009 People’s Republic of China

**Keywords:** Cancer, Metastasis, Organ-specific metastasis, Exosome, Tumor microenvironment, Therapy

## Abstract

Exosomes are cell-derived vesicles of 30 to 150 nm that contain diverse proteins, nucleic acids, and lipids. These vesicles facilitate effective intercellular communication and trigger profound environmental changes. In recent years, many studies have identified diverse roles for exosomes in tumor metastasis, a major cause of cancer-related deaths; furthermore, circulating tumor-derived exosomes can drive the initiation and progression of metastasis and determine the specific target organs affected. Fortunately, our growing understanding of exosomes and relevant modification technology have provided new ideas for potential treatment of tumor metastases. Here we review recent advances concerning the role of exosomes in metastasis, focusing on their regulatory mechanisms and therapeutic targeting in advanced cancer.

## Background

In most cases, primary cancers can be cured by surgical resection and adjuvant treatment. However, metastatic cancers are difficult to completely alleviate and are the cause of about 90% of cancer-related deaths [1]. In 1889, Stephen Paget proposed the “seed and soil” hypothesis of metastasis, which claims that the distribution of cancers is not random [2]. Current evidence shows that several processes occur during tumor metastasis, including angiogenesis and an epithelial-to-mesenchymal transition (EMT) [3]. Although the mechanisms of organ-specific metastasis are not fully understood, the roles of intercellular communication and the molecular characteristics of tumor cells are important considerations [4]. Before metastasis, the target organs are specially modified to establish a microenvironment suitable for tumor cell growth, known as a pre-metastatic niche (PMN). The establishment of a pre-metastatic niche involves vascular leakage, modification of the stroma and extracellular matrix, and immune system changes [5]. Also, paracrine interactions with stromal cells facilitate the formation of pre-metastatic niches and promote the growth of distant tumor cells [6, 7].

**Fig. 1:**
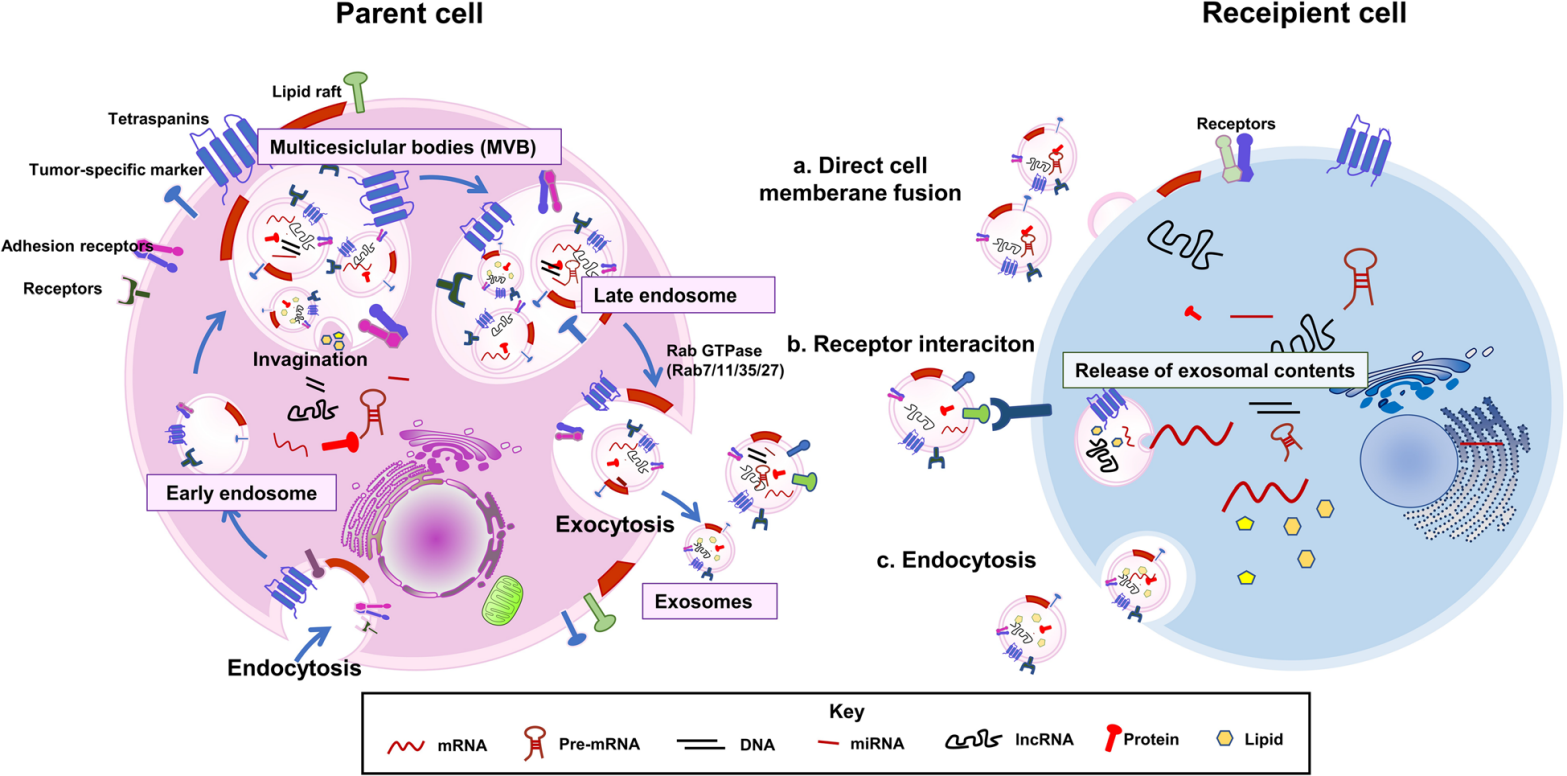
Biogenesis, secretion, and uptake of exosomes. Endocytosis often occurs at lipid rafts containing a variety of tumor-specific receptors and common membrane proteins, such as tetraspanins (eg, CD9, CD63, CD81), MHC I and II, and adhesion molecules (eg, integrins, cadherins), and results in the formation of early endosomes. Exosomes show inward budding of the multivesicular bodies (MVB). During this process, numerous proteins (e.g. receptor, ubiquitin-related proteins, heat shock proteins), nucleic acid (e.g. miRNAs, RNAs, DNAs, lnRNAs), transcriptional factors, and lipids (e.g., cholesterol, ceramide) can be selectively packed into MVB in a cell type-dependent manner. After early-to-late endosome conversion, late endosomes containing MVB fuse with the plasma membrane to secrete exosomes toward the extracellular space by exocytosis, which is mainly controlled by endosome-specific Rab GTPases, including Rab11/35, Rab7, and Rab27. The uptake of exosomes by recipient cells can be mediated by a) direct fusion of exosomes with the cell membrane of the recipients, b) by receptor-ligand interactions, or c) by endocytosis

Growing evidence suggests that exosomes and their contents contribute to the formation of the pre-metastatic microenvironment and to non-random metastasis patterns [8–10]. Exosomes are constitutively secreted by almost all cell types and can transport proteins, lipids, DNA, and RNA to recipient cells [11]. They contain many of the important raft lipids found in cellular membranes, such as ceramides, sphingolipids, cholesterol, and glycerophospholipids [12]. The principal RNA of exosomes is miRNA [13, 14], but they also contain long non-coding RNAs (LncRNAs), which regulate gene expression in many ways [15]. The biological characteristics of exosomes are summarized in Fig. 1.

Tumor exosomal miRNAs have been shown to interfere with the miRNA profile of target cells at a distance, thereby contributing to the formation of pre-metastatic niches [16, 17]. In addition, exosomes secreted from cells in secondary microenvironments may enhance brain metastases by producing CCL2 and recruiting IBA1-expressing myeloid cells [18]. Of note, metastasis-associated exosomes include not only tumor-derived exosomes but also exosomes released from other cells, such as T cells and fibroblasts [19]. In contrast to these metastasis-promoting effects, CD9 and CD82 proteins in exosomes have been shown to restrain tumor cell metastasis via communication with integrins [20]. In the following sections, we will highlight the latest findings on metastatic mechanisms mediated by exosomes, and discuss their implications for metastasis management.

### Exosomes regulate metastasis initiation and progression

**Fig. 2:**
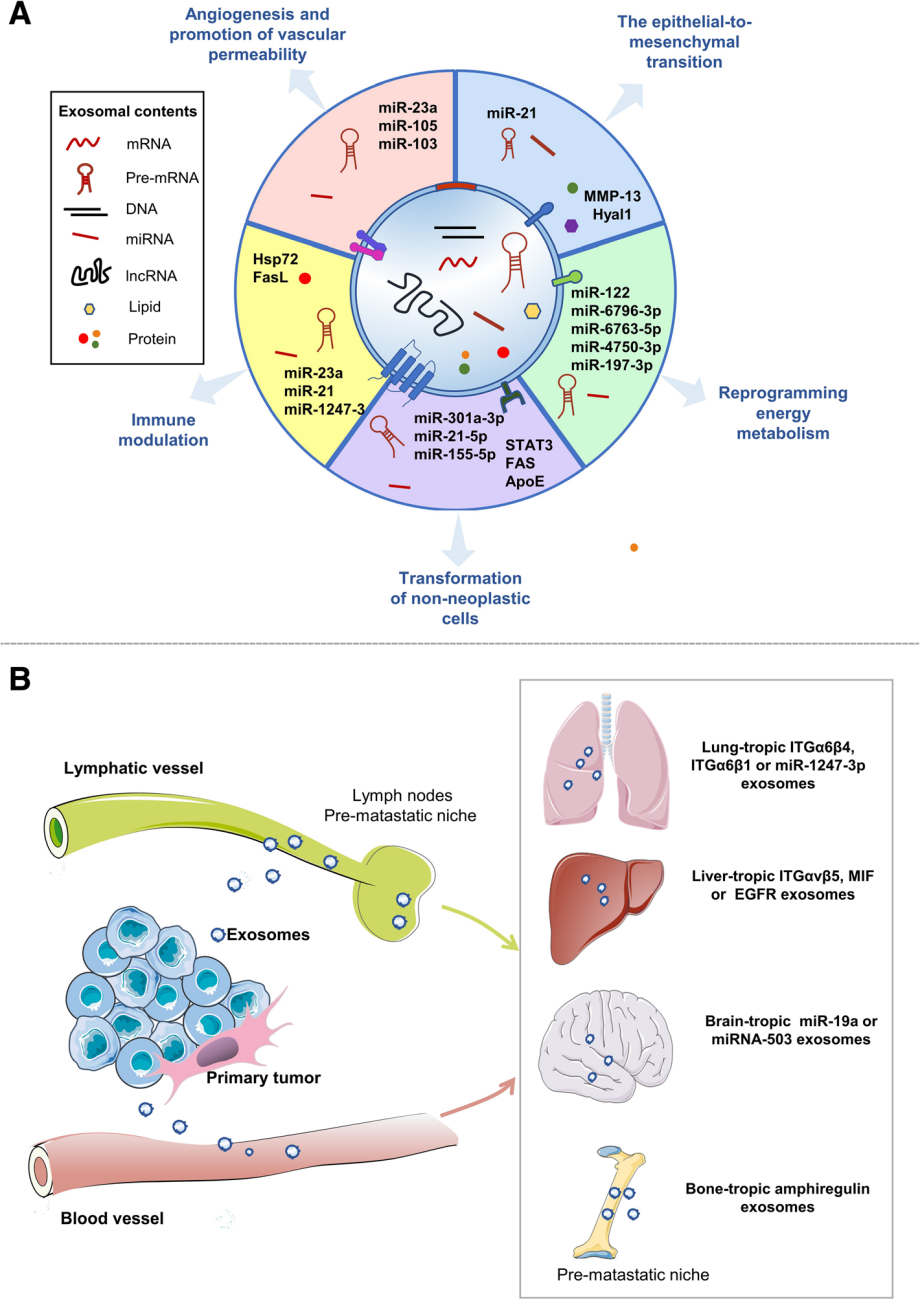
The role of exosomes in metastasis. **a** Exosomes regulate metastatic initiation and progression. Tumor-derived exosomes are involved in the epithelial-to-mesenchymal transition (EMT), angiogenesis, an increase of vascular permeability, alteration of the immune system, transformation of recipient cells, and reprogramming of energy metabolism. **b** The role of exosomes in organotropic metastasis. Primary tumor-derived exosomes can transfer proteins (e.g. integrin, EGFR) and nucleic acids (e.g. miRNAs, oncogenes) via blood vessels or lymphatic vessels to specific organs and bind to resident cells, thereby priming distant organ sites as pre-metastatic niches

Although only 0.01% of tumor cells in the blood stream can spread to distant sites, metastasis occurs frequently, and exosomes have been proposed to increase its success rate [21, 22]. Zomer et al. have reported that aggressive breast cancer cells promote the metastatic capacity of less-aggressive tumor cells, largely by transferring exosomes containing functional RNAs [23]. In the early stages of metastasis, exosomes participate in the sequential steps involved in both modulating tumor cells and establishing a distant pre-metastatic niche [24–26] (Fig. 2a).

#### The epithelial-to-mesenchymal transition (EMT)

EMT is a critical process in the initiation phase of metastasis. It is characterized by a loss of cell polarity and cell-cell adhesive ability in epithelial cells and an increase in the migratory and invasive ability that produces mesenchymal stem cells [27, 28]. Tumor-derived exosomes can promote the initiation and progression of metastasis by targeting EMT-related factors, such as transforming growth factor beta (TGFβ), caveolin-1, hypoxia-inducible factor 1 alpha (HIF-1α), and β-catenin [10]. McAtee et al. have shown that prostate tumor cells can increase the mobility of stromal cells by secreting exosomes rich in hyaluronidase Hyal1, a lysosomal hyaluronidase involved in prostate cancer metastasis [29], and Chen et al. have demonstrated that exosomes from highly metastatic cells can promote the migratory ability of low-metastatic cells. Mechanistically, the exosomes can trigger an EMT process via MAPK/ERK signaling [30]. Of note, the authors of two recent studies have reported that under hypoxic conditions, tumor cells can release exosomes enriched in miR-21 and matrix metalloproteinase-13 (MMP-13), which lead to an increase in vimentin and decrease in E-cadherin in normoxic cells, thus enhancing metastases occurring via EMT [31, 32]. With regard to the effect of a hypoxic microenvironment, the authors further discovered that exosomal MMP-13, operating via HIF-1α and HIF-1α, can directly regulate MMP-13. Nevertheless, it would be interesting to explore how hypoxic conditions affect exosome-mediated EMT.

#### Angiogenesis and promotion of vascular permeability

Compelling evidence shows that exosomes can deliver functional molecules to acceptor cells, thereby promoting angiogenesis and increasing vascular leakage [33, 34]. For example, one study has shown that exosomal miR-23a can induce angiogenesis in nasopharyngeal cancer [35]. In addition, Fang et al. have found that hepatoma cells generate exosomes rich in miR-103 that can promote tumor cell motility by increasing vessel permeability and targeting various endothelial junction proteins [36]. In a mouse model of melanoma, interactions between exosomes and the capillary wall have also been observed to increase vascular permeability, thereby leading to tumor cell leakage from blood vessels [37]. Similarly, tumor-derived exosomes carrying miR-105 can induce vascular permeability by specifically breaking tight junctions and natural barriers against metastasis, and tight junction protein ZO-1 has been shown to be the key target of exosomal miR-105 [17].

#### Immune modulation

In a variety of ways, exosomes can help metastatic cells to escape immune surveillance and induce a pre-metastatic microenvironment by transporting inflammatory factors [38]. In particular, tumor-derived exosomes expressing Hsp72 can restrain tumor immune surveillance by enhancing the activity of myeloid-derived suppressor cells [39]. In addition, exosomes released by breast cancer cells can decrease T-cell proliferation by targeting TGF-β [40]. It has been reported that tumor-derived exosomes expressing tumor antigens can inhibit T-cell activation and induce apoptosis of T-cells [41, 42]. Also, exosomes released from T cells have been shown to block the antitumor immune response by decreasing pMHC I expression in dendritic cells [43]. Furthermore, activated T-cell exosomes carrying bioactive FasL, a member of the tumor necrosis factor (TNF) family, may enhance the metastasis of melanoma and lung cancer cells by increasing the expression of MMP9 [44]. Andreola et al. have observed that tumor-derived exosomes expressing FasL can promote the apoptosis of lymphocytes [45]. Also, two studies have indicated that tumor-derived exosomes can block IL-2-mediated activation of NK cells and their cytotoxic activity [46, 47]. Furthermore, tumor-derived exosomes containing miR-23a may function as immunosuppressive factors by directly downregulating the expression of CD107a in NK cells [48].

More importantly, tumor-derived exosomes can precondition the tumor microenvironment for future metastasis by transporting inflammatory factors. For example, exosomes released from colorectal cancer cells are involved in formation of inflammatory pre-metastatic niches via the miR-21-TLR7-IL6 axis [49]. Furthermore, primary tumors can release exosomes carrying small nuclear RNAs to promote chemokine accumulation and neutrophil recruitment, thus aiding the formation of the pre-metastatic niche [50]. Interestingly, it has been found that TLR3 activation is crucial for metastasis but not essential for primary tumor growth; furthermore, the authors of this study confirmed that it is tumor-derived exosomal RNAs that activate TLR3, rather than tumor RNAs, indicating that the exosomal RNAs are selectively packed. These mechanisms need to be further explored [51]. Moreover, there may be other events that stimulate the formation of pre-metastatic niche, apart from TLR3 activation.

Exosomes also regulate the interaction between tumor cells and fibroblasts. Fibroblasts, in turn, produce exosomes containing pro-inflammatory cytokines that enhance tumor metastasis. For example, high-metastatic hepatocellular carcinoma cells release exosomes containing miR-1247-3p that target B4GALT3 and activate beta1-integrin-NF-kappaB signaling in fibroblasts, further boosting cancer progression by releasing pro-inflammatory cytokines, including IL-6 and IL-8 [52]. Exosomal IL-6 and IL-8 promote the local environmental changes that favor tumor metastasis.

#### Transformation of non-neoplastic cells

Tumor cells can affect recipient cells by releasing exosomes, thus promoting cancer metastasis, since exosomes can transfer molecules required for metastasis [53]. In particular, ovarian cancer cells can produce exosomes containing oncogenic proteins such as STAT3 and FAS, which increase the migratory ability of tumor cells [54]. Wang et al. have observed that pancreatic cancer cells can secrete exosomes containing miR-301a-3p to promote metastasis by inducing the M2 polarization of macrophages [55]. In addition, exosomes secreted from macrophages contribute to metastasis. For example, Lan et al. have discovered that M2 macrophages can release exosomes carrying miR-21-5p and miR-155-5p to promote colorectal cancer metastasis through the downregulation of BRG1 expression [56]. Similarly, M2 macrophages-secreted exosomes can promote the dissemination of gastric tumor cells by selectively transferring apolipoprotein E (ApoE), and ApoE can reshape cytoskeleton-supporting transportation by activating the PI3K-Akt signaling pathway [57].

#### Reprogramming energy metabolism

Metabolic adaptation allows cancer cells to adapt to an environment that lacks adequate nutrients [58, 59]. Recent studies have shown that tumor cells can alter their metabolic pattern to meet their energy needs under conditions of nutrient deprivation, giving them the ability to invade a hostile environment [60, 61]. In particular, exosomes function as metabolite carriers to promote tumor proliferation in nutrient-stressed microenvironments [62].

Extensive studies have shown that exosomal miRNAs can regulate the metabolic pathways associated with metastasis [63, 64]. For example, exosomes from CD105-positive renal cancer stem cells play a role in the formation of a premetastatic niche by transporting miRNAs. According to the enrichment analysis, miRNA takes the biggest proportion in the molecules affecting metabolic processes [65]. Furthermore, exosomal miR-122 s derived from breast cancer can modify the glucose metabolism of non-tumor cells in the premetastatic niche to promote metastasis. Mechanistically, tumor-derived exosomes carrying miR-122 can reduce glucose uptake via the downregulation of the glycolytic enzyme pyruvate kinase, thus increasing nutrient supplies [66]. More recently, Zhang et al. have found that exosomes released from pancreatic cancer cells can block the synthesis of GIP and GLP-1 in STC-1 cells in vitro by targeting PCSK1/3. These authors proposed that the miRNAs, including miR-6796-3p, miR-6763-5p, miR-4750-3p, and miR-197-3p, are critical for the process, although there may also be other undiscovered mechanisms involved [67]. In addition, exosomal miRNA-regulated metabolic reprogramming is a key mechanism that allows herpesviruses to form a tumor microenvironment. Also, exosomes from Kaposi’s sarcoma-associated herpesvirus (KSHV)-infected cells can specifically transport viral miRNAs to nearby cells, ultimately causing a metabolic switch toward aerobic glycolysis in the recipient cells [68].

### Organotropic metastasis

Metastasis is an organ-specific process in animal models that is not only dependent on the pattern of the vasculature or lymphatic vessels, but also on the tumor cell characteristics and host factors involved [69]. Many studies investigating the mechanisms of organ-specific metastasis have highlighted the roles of tumor cell traits, including the genes and pathways involved in the regulation of organotropism [17, 70–74]. Moreover, Fais and his peers have reported that microenvironmental pH is one of the factors influencing metastatic sites, since exosome fusion and interactions with the acceptor cells are affected by the microenvironmental pH [75, 76]. In the following sections, we will focus on the role of exosomes in organ-specific metastasis (Fig. 2b).

#### Lung and liver metastasis

It has been recognized that tumor-derived exosomes are involved in lung and liver metastasis. Initially, Hoshino et al. profiled a series of integrins expressed on tumor-derived exosomes because these integrins regulate the adhesion of exosomes to special tumor cell types and extracellular matrix (ECM) molecules in specific organs. Notably, they found that exosomes containing ITGαvβ5 specifically bind to Kupffer cells, thereby facilitating liver tropism, whereas exosomes expressing ITGα6β4 and ITGα6β1 favorably bind to lung-resident fibroblasts and epithelial cells, mediating lung tropism [8]. Also, Liu et al. have discovered that lung epithelial cells can sense tumor exosomal RNAs and be activated to recruit neutrophils by upregulating Toll-like receptor 3 (TLR3), thus facilitating the formation of a pre-metastatic niche [50]. However, neutrophils can also block metastasis [77], so it is necessary to identify the behavior of neutrophils at different stages and under different conditions. In addition, a recent study has revealed the mechanisms responsible for the lung metastasis of liver cancer: High-metastatic hepatocellular carcinoma cells can release exosomes containing miR-1247-3p to activate fibroblasts by targeting B4GALT3, and these activated fibroblasts can then release pro-inflammatory factors to boost lung metastasis [52]; this study has revealed a new molecular mechanism underlying the interaction between exosomal miRNAs and fibroblasts to promote lung metastasis.

Moreover, Costa-Silva et al. have reported that exosomes from pancreatic cancer can prime a liver pre-metastatic niche in the liver, with the exosomes enabling the Kupffer cells to alter growth factor β production and increase fibronectin secretion of hepatic stellate cells, thus shaping a fibrotic microenvironment with bone marrow-derived macrophages that facilitates metastasis. These authors further identified an exosomal protein, macrophage migration inhibitory factor (MIF), as an essential trigger of the pre-metastatic niche in the liver [78]. Although the study showed an important pattern of exosome-mediated metastasis, it remains unclear whether other components contained within exosomes of pancreatic cancer are needed to shape the liver’s pre-metastatic niche. Furthermore, other authors have reported that exosomes from highly malignant pancreatic tumor cells can promote the establishment of a liver pre-metastatic niche [79]; they found 79 exosomal proteins that are differentially expressed between highly metastatic and less metastatic cells, but they did not extensively research the mechanisms involved. It has also been proposed that exosomes derived from gastric tumor cells promote the formation of a liver-like microenvironment and enhance liver-specific metastasis. Exosomes are known to deliver EGFR to the liver to upregulate hepatocyte growth factor [80]. Most recently, Shao Y et al. have found that exosomes released from colorectal cancer cells can be specifically delivered to liver tissue and increase liver metastasis. In addition, these researchers reported that exosomal miR-21 is a key factor in establishing an inflammatory premetastatic niche [49], a finding that provides a rationale for targeting certain components of exosomes.

#### Brain metastasis

In the case of organ-tropic metastasis to the brain, several studies have reported a few brain-derived factors, such as secretory proteins and exosomal miRNAs, that alter the brain microenvironment to promote the colonization of brain metastasis [66, 81]. Camacho et al. have shown that exosomes derived from brain metastatic cells can transport metastasis-related proteins and miRNAs to non-brain metastatic cells, leading to increased cell-adhesive and invasive capability [82]. Zhang and colleagues have found that both human and mouse tumor cells with a normal expression of PTEN, an important tumor suppressor, have a decreased PTEN expression level after moving into the brain. Furthermore, they observed that the expression of PTEN in these brain metastatic tumor cells was restored after they were removed from the brain microenvironment. Finally, they showed that astrocytes can produce exosomes containing miR-19a, which decreases the expression of PTEN in circulating tumor cells in the brain microenvironment, thus leading to metastasis [18]. Astrocytes are a main stromal cell type in the brain, and their abnormal increase is associated with brain metastases in animal models and human patients [83]. These findings reveal that tumor cells can be remolded by their specific metastatic microenvironment, which to a large extent depends on exosomes. Similarly, a recent study on breast cancer has revealed that exosomal miRNA-503 can block T-cell production by increasing immune-suppressive cytokines, and this inhibition was only observed in brain metastasis [84].

#### Bone metastasis

Tumor-derived exosomes participate in bone metastasis by affecting bone metabolism, since tumor-derived exosomes can destroy the cycle of bone remodeling that occurs during the onset of a bone lesion [85], and exosomal miRNAs may be important regulators of bone metastasis [86]. Interestingly, Valencia et al. have found that exosomes carrying miR-192 can lessen bone metastasis [87], but they did not uncover the underlying mechanisms involved. In addition, Hashimoto et al. have identified eight miRNAs that are highly expressed in exosomes from prostate cancer cells with metastatic potential to bone, further demonstrating that exosomal hsa-miR-940 can enhance the osteoblastic phenotype of a bone metastatic microenvironment by targeting ARHGAP1 and FAM134A [88]. The functions of the remaining seven exosomal miRNAs are still unclear. Karlsson et al. have compared the exosomes from prostate tumor cells and fibroblastic cells and found that the exosomes from cancer cells can remarkably affect osteoclast formation by inhibiting the maturation of monocytic osteoclast precursors, which may represent a exosome-regulated abnormal formation of bone cells at the metastatic site [89]. Despite these revealing studies, the process of bone metastasis still needs to be studied in greater detail.

### Implications of exosomes in metastatic cancer therapy

There are biological vulnerabilities for tumor cells at various stages of metastasis, and these vulnerabilities provide opportunities for blocking the metastatic process. Because exosomes are involved in metastatic cascades and possess appealing properties for therapeutic delivery [90], we will comprehensively review below the current exosome-based therapeutic approaches with promising clinical applications.

#### Decreasing production and uptake of exosomes

Blocking secretion and uptake of exosomes is a potential approach for inhibiting metastasis. Preclinical and clinical studies have suggested that metastatic cancers can be at least partly alleviated by targeting the heparanase/syndecan-1 axis [91] or syndecan heparan sulfate proteoglycans, which are involved in the formation of exosomes and tumor cell dissemination [92, 93]. For instance, Sento et al. have found that heparin can remarkably block metastasis by decreasing uptake of tumor-derived exosomes in oral squamous carcinoma [94]. Furthermore, Nishida-Aoki and colleagues have designed a therapeutic antibody targeted at reducing the production of tumor-derived exosomes, leading to a decrease in distant metastasis of breast cancer in a mouse model [95]. These findings indicate that therapeutic antibodies targeting metastasis-related exosomes constitute a potential treatment approach. Also, targeted antibodies have been reported to significantly reduce cancer metastasis by promoting elimination of tumor-derived exosomes by macrophages [95]. In addition, a novel device has been designed to block or divert the spread of tumor cells: Creating an artificial pre-metastatic niche by implanting tumor exosomes in a 3D scaffold and then transplanting the scaffold into the peritoneal cavity of a mouse has led to the capture of ovarian tumor cells within the peritoneum and a redirecting of the tumor cells from their original target sites. This approach remarkably suppressed tumor metastasis at a distance [96]. However, this therapeutic strategy still needs to be tested in further in vivo experiments [22].

Increasing evidence has shown that extracellular acidity may influence the generation of exosomes by cancer cells. Initially, melanoma cells cultured under acidic conditions (pH 6.7) were found to secrete a larger number of exosomes than did the same cells cultured under physiological conditions (pH 7.4) [75, 97]. This phenomenon was later confirmed in other human tumor types, such as prostate cancer, melanoma, osteosarcoma, colon cancer, and breast cancer [76]. The mechanisms responsible for the increased secretion of exosomes in acidic environments are incompletely characterized, although some researchers have pointed out that producing more exosomes under low-pH conditions may be a way of relieving the intracellular accumulation of toxics material [76]. Based on this theory, comparable proton-pump inhibitors have been developed to decrease plasmatic exosome levels in xenograft models [98]. Thus, the alkalinizing approach may be a potential anti-tumor strategy for patients with tumor metastasis [85].

#### Exosomal miRNAs

Exosomes contain a variety of miRNA that can be targeted to restrain exosome-mediated metastasis [99]. In colorectal cancer (CRC), CRC-derived exosomal miRNAs have been shown to promote tumor cell proliferation [90, 100], making it possible to use gene therapy to suppress tumor metastasis by engineering exosomal miRNA. Furthermore, miR-379 in CRC-derived exosomes has been found to down-regulate the migration of CRC cells, and transfer of these engineered miR-379-overexpressing exosomes to recipient cells reduced their migration [101]. Similarly, Zaharie et al. have demonstrated that exosomal microRNA-375 inhibits tumor cell dissemination by blocking Bcl-2 in colon cancer, suggesting that exosomal microRNA-375 can be considered a potential therapeutic target [102]. Furthermore, it has been suggested since there is a positive correlation between exosomal miR-193a expression and liver metastasis of colon cancer, the major vault protein (MVP) can possibly decrease the level of circulating exosomal miR-193a, providing a new therapeutic approach for metastatic colon cancer [103].

#### Vaccination

Overwhelming evidence demonstrates the immune-suppressive function of exosomes. For instance, they can inhibit the cytotoxicity of NK cells and differentiation of dendritic cells (DC), as well as induce apoptosis of cytotoxic T-cells and M2 polarization of macrophages, all of which promote tumor metastasis [9, 24]. In the first phase I trial of exosome therapy, among 15 metastatic melanoma patients receiving exosome vaccinations, only WHO grade II toxicity was present and one patient exhibited a partial response, suggesting that it is feasible to produce large-scale exosomes for therapeutic purpose because of their good safety profiles [104]. Notably, in the second Phase I trial performed at Duke University in advanced non-small cell lung cancer, a high efficacy of autologous dendritic cell (DC)-derived exosomes and MHC class I peptides led to long-term survival [105].

#### Exosomes as promising delivery systems

Given their high physicochemical stability and biocompatibility, exosomes can be chemically or biologically modified to yield delivery systems that can enhance the treatment outcomes of chemotherapeutic agents, as well as reduce drug toxicity [106–110]. One study has found that exosomes can be used to deliver doxorubicin specifically to tumor tissues through intravenous injection, decreasing tumor growth without overt toxicity [111]. In addition, Ohno et al. have shown that exosomes can efficiently carry let-7a miRNA to EGFR-expressing breast cancer cells in animal models [112]. Genetically engineered exosomes have also been shown to inhibit schwannoma tumor progression by delivering suicide mRNAs and proteins [113]. Finally, research in zebrafish has demonstrated that exosomes can increase the uptake of drugs in the brain, demonstrating their capacity to function as delivery systems for anticancer drugs targeting brain metastasis [114].

## Conclusions

In conclusion, exosomes act as an important regulator in metastatic cascades, including the initiation, progression, and colonization of metastasis in distant organs, by delivering functional molecules and directly affecting target cells. Undoubtedly, targeting exosomes associated with metastasis represents a new approach to the development of novel, effective anti-tumor therapeutic agents.

There are many challenges in the field of exosomes, and many worthwhile directions for future studies to pursue. First, it is important to develop insights that will allow for a standardization of the classification of exosomes. Among the challenges to be met: 1) More efficient methods and reproducible criteria are needed for isolating and characterizing pure groups of specific subtypes of exosomes according to their biophysical and biological features. 2) For different subtypes of exosomes, their specific target cells and the consequences that ensue after they reach acceptor cells need to be further explored. 3) the process of exosome uptake and cargo delivery into the cytosol of receptor cells need to be more fully understood [115].

Second, the tumor microenvironment is linked to the progression of various stages of tumors [116]. It will be important to explore how components of the tumor microenvironment affect exosome behavior [117, 118]. It is also pivotal to learn more about the roles of exosomes in metabolic changes related to metastasis [58]. For example, Abhinav and colleagues have devised an experimental and computational platform to analyze exosomes with different metabolite components in various environments, which is helpful for understanding the metabolic alterations in tumor microenvironment components; however, the platform still needs to be optimized [119]. Consequently, it will be interesting to determine whether exosomes can be used as markers for disease progression and treatment [120–122]. Third, the precise roles of exosomes in organ metastasis need to be further defined. The knowledge of which component of exosomes is responsible for tissue specific targeting, local environment modification, and possibly immune alteration should be investigated. In particular, the mechanism involved in brain metastasis should be a research focus because of the unique repercussions of the blood-brain barrier for cancer and the potential for future therapeutic development. Recently, He C et al. have proposed that RNAs are responsible for the major functions of exosomes, a conclusion that suggests new directions for research into the functional contents of exosomes [123].

Although therapeutics based on exosomes are promising, there are several significant issues that must be addressed before exosomes can be tested clinically: 1) Standard guidelines for the manufacture, purification, storage, usage, duration, and dosage of exosome-based drugs remain to be established, since it is reported that surgical intervention may trigger local hypoxia and inflammatory response, both of which are relevant to metastasis [124–127]. 2) It must be verified that the sources of the exosomes are safe for clinical use [75]. 3) How the acceptor cells can be protected by removing any carcinogenic components of the exosomes must be investigated [128]. 4) How interactions between therapeutic exosomes and unexpected cells can be avoided must also be examined [129]. 5) Is it feasible that only autologous exosomes be used? Under what circumstances can heterologous be explored? 6) Is it promising to design exosome-based drugs targeting the hallmarks of cancer metabolism [130]? 7) Since many studies have focused on the application of exosome-based vaccines as alternative approaches to suppress tumor growth [131], is it meaningful to develop exosome-based vaccines to prevent metastasis? Significant progress has been made in characterizing the role of exosomes in tumor metastasis, and the therapeutic potential of using exosomes or their derived vesicles is entering an uncertain but exciting stage.

## Abbreviations

B4GALT3: Beta-1,4-galactosyltransferase 3
BRG1: ATP-dependent chromatin remodeler SMARCA4
CCL2: Chemokine (C-C motif) ligand 2
c-MET: Tyrosine-protein kinase Met
CRC: Colorectal cancer
DC: Dendritic cells
ECM: Extracellular matrix
EGFR: Epidermal growth factor receptor
EMT: Epithelial to mesenchymal transition
ERK: Extracellular signal-regulated kinases
FAS: Fatty acid synthase
FasL: Fas ligand
GIP: Glucose-dependent insulinotropic peptide
GLP-1: Glucagon-like peptide-1
HIF-1α: Hypoxia-inducible factor 1 alpha
IBA1: Ionized calcium-binding adapter molecule 1
IL-2: Interleukin-2
KSHV: Kaposi’s sarcoma-associated herpesvirus
LncRNAs: Long non-coding RNAs
M2: macrophages Alternatively activated macrophages
MAPK: Mitogen-activated protein kinases
MIF: Migration inhibitory factor
MMP-13: Matrix metalloproteinases-13
MMP9: Matrix metallopeptidase 9
MVP: Major vault protein
NK: Natural killer
NSCLC: Non-small cell lung cancer
PCSK1/3: Proprotein convertase subtilisin/kexin type 1/3
pMHC I: Peptide-MHC-I complexes
PMNs: Pre-metastatic niches
PTEN: Phosphatase and tensin homolog
STAT3: Signal transducer and activator of transcription 3
TGFβ: Transforming growth factor beta
TLR3: Toll-like receptor 3
TNF: Tumor necrosis factor
